# Processing Coordinate Structures in Chinese: Evidence from Eye Movements

**DOI:** 10.1371/journal.pone.0035517

**Published:** 2012-04-25

**Authors:** Chen Qingrong, Huang Yan

**Affiliations:** 1 Department of Psychology, Nanjing Normal University, Nanjing, China; 2 Department of Applied Foreign Language Studies, Nanjing University, Nanjing, China; University of Leicester, United Kingdom

## Abstract

This article reports the results of an eye-tracking experiment that investigated the processing of coordinate structures in Chinese sentence comprehension. The study tracked the eye movements of native Chinese readers as they read sentences consisting of two independent clauses connected by the word *huo zhe*. The data strongly confirmed readers' preference for an initial noun phrase (NP)-coordination parsing in Chinese coordination structure. When *huo zhe* was absent from the beginning of a sentence, we identified a cost associated with abandoning the NP-coordination analysis, which was evident with regard to the second NP when the coordination was unambiguous. Otherwise, this cost was evident with regard to the verb, the syntactically disambiguating region, when the coordination was ambiguous. However, the presence of a sentence-initial *huo zhe* reduced reading times and regressions in the *huo zhe* NP and the verb regions. We believe that the word *huo zhe* at the beginning of a sentence helps the reader predict that the sentence contains a parallel structure. Before the corresponding phrases appear, the readers can use the word *huo zhe* and the language structure thereafter to predicatively construct the syntactic structure. Such predictive capability can eliminate the reader's preference for NP-coordination analysis. Implications for top-down parsing theory and models of initial syntactic analysis and reanalysis are discussed.

## Introduction

Research has provided conflicting evidence regarding how people process sentences. Two major parsing approaches have been identified: top-down and bottom-up. In top-down parsing, readers are able to build sentence structure using grammatical information to construct a representation of a sentence's syntactic structure before encountering linguistic input [1], [2], [3], [4]. This view contrasts with the assertions of the bottom-up approach. Following the principle of gradual integration based on lexical input, bottom-up syntactic analysis places nodes into phrase markers through which the related child nodes contribute to the construction of high-level nodes. A number of researchers have proposed that a syntactic structure is projected from the phrase heads [5], [6], [7]. According to this view, the syntactic parser must wait until the head of a phrase emerges before attaching any other material that is part of the phrase. However, a considerable body of research does not support this view; the findings indicate that even when the head of a phrase does not appear, the reader still makes a number of decisions regarding sentence processing without delaying syntactic judgment and the construction of related structures [8], [9], [10], [11].

To apply a top-down strategy, a parser must be able to build syntactic structure before encountering any of the lexical input necessary to construct this structure. Chen et al. argued that a storage cost is associated with maintaining syntactic predictions [12]. They tested the comprehension of sentence pairs similar to the following (the critical region is in italics):

1a. The claim alleging that *the cop who the mobster attacked* ignored the informant might have affected the jury.
1b. The claim which *the cop who the mobster attacked* ignored might have affected the jury.

According to the top-down storage cost hypothesis, the critical region is processed more rapidly in 1a than in 1b. In both 1a and 1b, a verb is predicted by the noun phrase (NP), *the claim*. The second sentence, 1b, includes the added prediction of a position to be associated with the wh-filler, *which*. When readers process this region of the latter sentence, they must maintain in their memory a prediction of a *trace* corresponding to the relative pronoun *which*. This hypothesis was supported by results showing that participants read the critical region in 1b more slowly than the same region in 1a, which suggests that keeping track of a wh-filler has a processing cost. Based on these findings, Chen et al. claimed that maintaining syntactic predictions in memory has a processing cost, with more predictions corresponding to slower reading. Nakatani et al. (2008) used a self-paced reading paradigm to examine syntactic expectation costs in Japanese sentence comprehension [13]. They controlled the number of dependents of an upcoming verb by manipulating the presence/absence of a locative postpositional phrase modifier of the verb and the presence/absence of a dative argument of the verb. The results indicated a measurable expectation cost when an additional verb and complementizer were expected.

A number of studies have argued against the top-down storage cost hypothesis. Wright and Garrett (1984) designed two lexical decision experiments exploring the effect of preceding syntactic information [14]. In both experiments, the syntactic category of the target word was either predictable (e.g., The interesting clock seems very *tolerable*) or legal but unpredictable (e.g., Your visiting friend should enjoy *tolerable*). The results revealed that the lexical decision latency was reduced significantly when the target word's category was predictable based on the preceding context. In contrast with previously mentioned studies that focused on a single word, the present research explores whether the processing of complex phrasal or clausal structures is facilitated when the syntactic structure is predictable. This research interest is shared with Altmann, van Nice, Garnhan, and Henstra (1998), who examined the effect of context through four eye-movement studies [15]. In their studies, the following sentence was considered: “*She'll implement the plan she proposed tomorrow, they hope*.” Due to the preference of Late Closure, readers of this sentence were likely to attach the incoming material (i.e., tomorrow) to the phrase currently being processed (i.e., she proposed). This attachment renders a garden path due to the temporal mismatch between the adverb (*tomorrow*) and the verb (*proposed*). Thus, English readers find it easier to process a sentence when this low attachment is correct (e.g., *She'll implement the plan she proposed last week, of course*) than one in which high attachment is forced (e.g., *She'll implement the plan she proposed next week, of course*). However, Altmann et al. found that when the target sentence was preceded by a context sentence that explicitly directed attention toward the high predicate (e.g., *When will Fiona implement the plan she proposed?*), this preference was eliminated. Altmann et al. suggested that readers could predictably activate the adverbial representation in advance and use it to integrate the subsequent information. We agree that this interpretation is plausible. Previous studies have found that in Chinese, context can promote the processing of words, text and ambiguous sentences [16], [17], [18]. Compared to English, context may play a more important role in sentence processing in Chinese [19].

However, a number of researchers question whether syntactic prediction is specifically induced by the preceding context sentence [20], [21]. Many researchers believe that this predictable syntactic information will be realised by other structures. Frazier and Clifton (2000) used a self-paced reading paradigm to examine the effect of *either* when it was separated from the disjunction over which it has scope, such as, *Mary is looking either for a maid or a cook* and *Sam either wants his mother or his father*. The results showed that the presence of *either* increases the predictability of the final coordination and facilitates its processing. In Frazier et al.'s study, the effect of *either* was found in the final region. Therefore, the effect was likely related to clause wrap-up [22], [23]. In addition, slow reading time, which was due to the self-paced reading paradigm that was used, tends to magnify any predictive processes involved in normal reading [24]. To rule out these explanations, Staub and Clifton (2006) used an eye-tracking paradigm to examine whether the garden path effect could be eliminated or reduced when the syntactic structure was predictable. Their study examined the effect of syntactic prediction by monitoring readers' eye movements as they read sentences containing two noun phrases or two independent clauses connected by the word *or*, as shown in Examples 2a–2d:

2a. John borrowed a rake or his wife bought one.
2b. Either John borrowed a rake or his wife bought one.
2c. My friend wrote a short story or an essay in the school magazine.
2d. My friend wrote either a short story or an essay in the school magazine.

Examples 2a and 2b contain an S-coordination structure in which two independent clauses are connected by the word *or*. In contrast, Examples 2c and 2d contain an NP-coordination structure in which two noun phrases are joined by the word *or*. The results showed a general speed increase in reading the words after *or* in both the NP-coordination and S-coordination sentences with the sentence-initial *either*. In the absence of *either*, readers misanalysed the S-coordination structure as an NP-coordination structure, and the downstream garden path effect was evident. However, this effect disappeared when *either* was present. The results support the top-down strategy that parsers can build predictable syntactic structures. This predictive information facilitated the processing of the coordinate structure and enabled readers to avoid the implausible NP-coordination analysis in the S-coordination sentences. Significantly, in the study by Staub et al. (2006), the NP-coordination analysis of the S-coordination sentences was always implausible (e.g., *Linda bought the red car or her husband leased…*). In a relevant experiment, Staub (2007) used S-coordination sentences in which the NP-coordination analysis was plausible but could be eliminated with the presence of a comma in two conditions, such as, *The boys will use the skis (,) or the sled will make….*. The critical finding was the presence of a garden path effect in the disambiguating region in the *no comma* condition. This effect was found on both first pass time and go-past time, and the presence of *either* did not eliminate the garden path effect. In fact, the garden path effect was numerically smaller when *either* was absent compared to when *either* was present (366 ms vs. 368 ms), which was interpreted in terms of the initial syntactic analysis and the reanalysis [25]. Regardless of whether the word *either* was present or absent, readers adopted the NP-coordination analysis in the ambiguous region, and yet this analysis was abandoned when the S-coordination was confirmed. This indicates that the probability of the NP-coordination analysis occurring was not reduced by the presence of *either*.

Two questions emerge. Does syntactic predictability help readers build predictable structure and reduce or eliminate the garden effect in Chinese sentence comprehension? Is the NP-coordination analysis preferred when readers process coordinate structures in Chinese? This study aims to answer these two questions. In considering the first question, Hsiao and Gibson (2003) used a self-paced reading paradigm to explore the processing of Chinese relative clauses, as illustrated in Examples 3a–3d [26].

3a. Chinese singly-embedded object-extracted relative clause fuhao yaoching de guanyuan shinhuaibugui danshi shanyu yintsang
  N1 V1 de1 N2 ….
  The official who the tycoon invited has bad intentions but is good at hiding them.
3b. Chinese singly-embedded subject-extracted relative clause yaoching fuhao de guanyuan shinhuaibugui danshi shanyu yintsang
  V1 N1 de1 N2 ….
  The official who invited the tycoon has bad intentions but is good at hiding them.
3c. Chinese doubly-embedded object-extracted relative clause fuhao yaoching de faguan gojie de guanyuan shinhuaibugui
  N1 V1 de1 N2 V2 de2 N3 ….
  The official who the judge who the tycoon invited conspired with has bad intentions.
3d. Chinese doubly-embedded subject-extracted relative clause yaoching gojie faguan de fuhao de guanyuan shinhuaibugui
  V1 V2 N1 de1 N2 de2 N3 ….
  The official who invited the tycoon who conspired with the judge has had intentions.

The critical regions of comparison in the singly-embedded versions consisted of the first three words: N1 V1 de (for object-relative clauses) or V1 N1 de (for subject-relative clauses). The critical regions in the doubly-embedded versions consisted of six words: N1 V1 de1 N2 V2 de2 (for object-relative clauses) or V1 V2 N1 de1 N2 de2 (for subject-relative clauses). The results showed that object-extracted relative clause structures were less complex than the corresponding subject-extracted structures in both singly and doubly-embedded Chinese relative clauses. Hsiao and Gibson (2003) suggested that, “the results follow from a resource-based theory of sentence complexity, according to which there is a storage cost associated with predicting syntactic heads in order to form a grammatical sentence” (p. 3). They argue that Chinese readers can predict the appearance of a relative clause when they process the first verb (i.e., yaoching, “invite”) in the subject-relative structure, given that the verb does not have a subject. Thus, three syntactic heads are necessary: a main verb for the sentence together with the relative clause genitive marker (*de*) and a NP object for the verb in the relative clause. After the noun object (i.e., fuhao, “tycoon”) is processed, two syntactic heads are still needed: the main verb and the relative clause genitive marker. Processing the object-relative structure requires fewer predicted heads at each of these positions. For example, after processing the first word (i.e., fuhao, “tycoon”) in the object-extraction structure, only a single head is predicted: a verb for the clause because this could be the main clause. After the next word (i.e., yaoching, “invite”) is processed, again only one head is predicted: a noun object of the verb. Therefore, processing Chinese subject-relative structures requires more storage resources than processing Chinese object-relative structures. We believe that Hsiao et al. may be correct in arguing that maintaining syntactic prediction in Chinese has a processing cost. In addition to the findings showing normal Chinese readers experience greater difficulty in processing subject-relative structures than object-relative structures [27], [28], [29], similar reports have been obtained from Chinese aphasic speakers [30], [31], [32].

However, we note that syntactic prediction is also likely to produce a facilitative effect in Chinese. Rayner et al. (2005) performed an eye-tracking experiment to examine the effect of word predictability in Chinese [33]. The predictability of the target words from the preceding context was high, medium, or low. The results showed that readers fixated for less time on high- and medium-predictable target words than on low-predictable target words. Rayner et al. suggested that, “Chinese readers, like readers of English, exploit target word predictability during reading” (p. 1089). Wu and Shu (2002) used a character decision task to examine the effect of sentence context on ambiguous Chinese words [34]. The results showed that lexical decision time was shorter when the meaning of a target word was consistent with the sentence context. In the present experiment, we intend to further explore whether syntactic prediction reduces or even eliminates the garden path effect associated with complex clause structure, rather than focusing only on single characters or words in Chinese.

The second question concerns whether the NP-coordination analysis is preferred when readers process coordinate structures in Chinese. In English, *or* is analysed as a coordinator between two clauses, verb phrases, or noun phrases when *either* is present in the sentence initially [25], [35], [36]. In modern Chinese, the word *huo zhe* (used as “or” in English) is the marker of parallel structure that connects two language materials of similar structure in the sentence. It can be used to connect noun phrases (NP; for example, 男孩子或者女孩子都可以, Boys or girls are able to), verb phrases (VP; for example, 升学或者参加工作由你自己决定, It is up to you to choose to keep studying or to work), and sentences (for example, 你们春节到我家里来过或者我们一起外出旅行, You can come to our house to celebrate Chinese New Year, or we can travel together). If the first *huo zhe* (commonly used as “either” in English) connects a VP, the second *huo zhe* also has to connect a VP (for example, 或者问你或者问我都可以, It is either fine to ask him or to ask me). If the first *huo zhe* connects a clause, the second *huo zhe* must also connect a clause (for example, 或者球员更换球队或者经纪人说服经理给他加工资, Either the player will change teams, or the agent will convince the team manager to increase his salary). Thus, Chinese readers do not adopt the NP-coordination analysis when processing sentences such as, “*Either the boys will use the skis or the sled*”. We predict that Chinese readers prefer NP-coordination analysis, which is shown in Figure S1 (a). When the word *huo zhe* appears at the beginning of a sentence, the readers are able to predict that the sentence will contain a parallel structure (for example, VP or VP, NP or NP, S or S). Readers can use the language material structure connected with the word *huo zhe* to predicatively construct the sentence structure, as shown in Figure S1 (b).

Before presenting the details of the experiment, we note that Chen et al. (2010) performed an experiment similar in some respects to the one we present here [37]. In that study, participants read sentences such as Examples 4a–4d:

4a. Sentence with an initial *huo zhe* and temporary syntactic ambiguity
  或者球员更换球队或者经纪人说服经理给他加工
  Either the player changes teams or the agent convinces the team managers to increase his salary.
4b. Sentence with an initial *huo zhe* but without temporary syntactic ambiguity
  或者厂长补发工资或者工程师拒绝继续签新的合同
  Either the factory manager retroactively pays the unpaid salary or the engineers refuse to sign a new contract.
4c. Sentence without an initial *huo zhe* but with temporary syntactic ambiguity
  警察找到物证或者目击者愿意为受害者当人证
  The police found physical evidence or the witnesses are willing to testify for the victims.
4d. Sentence without an initial *huo zhe* and temporary syntactic ambiguity
  叶林购买轿车或者她丈夫租借一辆车子给她用
  Ye Lin will buy a car or her husband will rent one for her.

Chen et al. found significantly longer reading time and more regressions in the 4c condition than in any other condition. They suggested that this effect was due to the use of NP-coordination analysis when readers processed the ambiguous region in Example 4c (p. 682). This interpretation cannot be confirmed because the four conditions in the study differ, not only in terms of the syntactic structures of the sentences but also in their semantic and referential meanings. Therefore, the factor that contributes to the difference cannot be clearly identified. Compared to the 4c condition, the noun phrase presented after *huo zhe* in the 4d condition was not a plausible object (e.g., goumai jiaoche huozhe *ta zhangfu*). If readers adopt the NP-coordination analysis in the ambiguous region, one should observe a larger effect of reading time and regressions induced by this implausible object in the 4c condition. However, this inference was inconsistent with their experimental data. In this study, readers spent more reading time (i.e., first fixation time, first pass time, and go-past time, with more regressions) in the 4c conditions than in the 4d conditions.

We believe that three explanations can account for the aforementioned results. First, the stimuli used in Chen et al.'s study suggest that the study did not have an appropriate design to convincingly address the issues of syntactic prediction and NP-coordination parsing preference. When reading the sentences in Examples 4a–4d, Chinese speakers can see that, apart from structural differences, the semantic and referential meanings of each sentence can also be quite different in varying conditions. This is not a typical design for a psycholinguistic study. This design creates the possibility that differences among conditions might be caused by other uncontrolled factors. This obvious flaw makes the four critical conditions incomparable. For example, in Chen et al (2010), the number of strokes was not controlled for across the four critical conditions. Therefore, the effect on reading time may be an effect of processing related to the number of strokes for the Chinese characters [38], [39], [40]. Second, in the 4d condition, the first NP was the subject of the first clause, and so the personal pronoun and noun very likely served as the subject of the second clause [41]. Thus, the personal pronoun in the 4d condition (e.g., *ta*) likely facilitated the processing of the ambiguous region [42], [43]. Finally, Chen et al. did not control the semantic relationship between the two noun phrases in the NP-huo zhe-NP string. This semantic relationship could influence the experimental results [44], [45], . To rule out the design faults and avoid misunderstandings of the Chinese coordination structure, we designed the present experiment using better controlled stimuli.

## Methods

### Ethics Statement

The procedures for this study have been approved by the Institutional Review Board of the Nanjing Normal University. Informed consent was obtained in written form from all participants.

### Participants

Sixty students participated in the experiment for course credit or payment. Before the experiment, all of the students provided informed consent. They were all native speakers of Mandarin Chinese with normal or corrected-to-normal vision and had no history of neurological or language impairments. The participants were not informed of the purpose of the experiment and had no previous exposure to the experimental items.

### Stimuli and Design

We constructed 12 pairs of sentences similar to those presented in Examples 5a and 5b. All of the experimental stimuli appear in the Materials S1.

5a. 或者校长资助*孤儿*/*或者其他人*/*组织*/起来共同资助他
  Either the headmaster supports the orphan/or other people/organise/together to support him.
5b. 或者厂长提高*待遇*/*或者工程师*/*拒绝*/继续签新的合同
  Either the factory director improves the treatment/or the engineers/refuse/to sign the new contract.

Both sentences began with a *huo zhe* and consisted of two independent clauses connected by another *huo zhe*. The only difference between 5a and 5b was that the NP following the second *huo zhe* served as a plausible direct object of the verb in the initial clause in 5a. Therefore, the garden effect would appear in this region [47]. In subsequent discussion, we refer to “5a” as the *huo zhe ambiguous S-coordination* and “5b” as the *huo zhe S-coordination*.

We also constructed 12 pairs of sentences similar to those presented in Examples 5c and 5d. To exclude the impact of different numbers of words, we conducted a phrase-judgment experiment. The results showed that the average accuracy rate was 98%. Under the ambiguous condition, the average response time was 457 ms (*SD* = 32 ms). Under the unambiguous condition, the average response time was 468 ms (*SD* = 44 ms). No significant difference was found between the two conditions (*t* (29) = −1.26, *p* = 0.218). The only difference between 5c–5d and 5a–5b was that the word *huo zhe* did not precede the entire sentence in 5c–5d. We refer to “5c” as the *ambiguous S-coordination* and “5d” as the *S-coordination*.

5c. 校长资助*孤儿*/*或者其他人*/*组织*/起来共同资助他
  The headmaster supports the orphan/or other people/organise/together to support him.
5d. 厂长提高*待遇*/*或者工程师*/*拒绝*/继续签新的合同
  The factory director improves the treatment/or the engineers/refuse/to sign the new contract.

We balanced the word frequency from a pool of 16,593 words, according to the Modern Chinese Frequency Dictionary (Institute of Language Teaching and Research, 1986) [48] and the stroke of the various regions of interest (the region is in italics) in 5a and 5b (e.g., the NP region (*gu er*), *huo zhe* NP region (*huo zhe qi ta ren*), and Verb region (*zu zhi*). In this pool (p. 1–490), the number of times that each word was used is ≥2. For *ambiguous S-coordination*, the mean frequencies in the NP, *huo zhe* NP, and Verb regions were 600 (*SD* = 400), 1450 (*SD* = 1290), and 590 (*SD* = 510) per one hundred thousand, respectively. For *S-coordination*, the corresponding values were 500 (*SD* = 300), 1270 (*SD* = 1100), and 580 (*SD* = 480) per one hundred thousand. For *ambiguous S-coordination*, the mean stroke in the NP, *huo zhe* NP, and Verb regions were 13.75 (*SD* = 3.08), 36 (*SD* = 6.62), and 18.5 (*SD* = 2.84), respectively. For *S-coordination*, the corresponding values were 11.92 (*SD* = 5.07), 36.91 (*SD* = 6.92), and 16.92 (*SD* = 3.55). The results showed that the differences in word frequency and stroke were not significant in any region (*t*s<1.3, *p*s>0.3).

Forty participants rated the rationality of the experimental sentences on a scale ranging from 1 to 5. Participants were instructed to assign a rating of 1 to sentences that were “very unreasonable” and to assign a rating of 5 to sentences that were “very rational”. The 24 sentences were intermixed with 50 fillers. The mean ratings for the different conditions were as follows: *huo zhe ambiguous S-coordination* (*M* = 4.15, *SD* = 0.41) and *huo zhe S-coordination* (*M* = 4.09, *SD* = 0.34). No significant difference was observed between conditions (*F*s<1, *p*>0.7).

Thirty participants rated the semantic relation between the noun in the NP region and another noun in the *huo zhe* NP region on a scale ranging from 1 to 5. Participants were instructed to assign a rating of 1 to the two nouns with “weak” semantic relations between them and a rating of 5 to those pairs with “strong” semantic relations. Each participant rated 24 noun pairs, 12 in the ambiguous condition and 12 in the unambiguous condition. The 24 noun pairs were intermixed with 24 fillers. None of the participants in this part of the study took part in the eye movement experiment. The mean ratings between the 2 nouns were 3.42 (*SD* = 0.52) and 3.25 (*SD* = 0.45) in the ambiguous and the unambiguous conditions, respectively. The difference was not significant (*t*<1, *p*>0.4).

Two variables were manipulated in a 2×2 within participant factorial design. For the eye-tracking experiment, the four conditions were the *huo zhe ambiguous S-coordination* (version 5a), *huo zhe S-coordination* (version 5b), *ambiguous S-coordination* (version 5c), and *S-coordination* (version 5d). Each condition had 12 sentences. The only difference between the first two conditions and the latter two conditions was in the presence or absence of the sentence-initial *huo zhe*. These sentences were divided into two lists to ensure that each participant saw only six sentences in each of the four conditions and one version of each sentence. The counterbalancing scheme aimed to achieve the following: (a) each participant read any particular sentence no more than once, and (b) sentences of each type in either list were equal in number.

### Procedure

Participants were tested individually. Eye movements were monitored with a SensoMotoric Instruments (SMI) iView Hi-Speed eye-tracker, sampling at 1250 Hz (tracking resolution <0.01°) from the right eye (viewing was binocular). A forehead rest and a bite-bar were used to stabilise participants' head position and to minimise signal interference caused by head movements. All sentences were displayed on a single line. Stimuli were displayed on a 17-in. monitor. Participants were seated 65 cm from the computer screen. At this distance, each Chinese character subtended a visual angle of 1.05°.

Upon arrival to the lab, participants were provided instructions. A 13-point calibration routine was performed, and its accuracy was checked after every fourth trial. Participants were instructed to read sentences silently for understanding at their normal rate. After reading each sentence, a yes/no comprehension question appeared and remained on the screen until a response was made. Participants did not receive feedback on their responses. Participants completed four practice trials before the main experimental block. The entire experiment lasted approximately 20 minutes.

## Results

The following four eye-movement measures were computed: first fixation duration, first pass time, go-past time, and percentage regressions [24]. First fixation duration refers to the duration of the first fixation in a region. First pass time is the sum of all fixations in a region prior to leaving the region for the first time, to move to either the left or the right of that region. The first two measures reflect early stages of processing such as lexical access [49]; however, syntactic misanalysis has also been shown to affect these measures [47]. Go-past time (sometimes called regression path duration) is the elapsed time from first fixation in the region until the reader leaves the region and moves to the right, including any time spent moving to the left of the region after a regressive eye movement and any time spent rereading material in the region before moving on [23], [50]. Finally, the percentage regressions measure includes only regressions made during the reader's first pass through the region; it does not include regressions made after re-fixation to the region [51]. This measure may reflect the processing difficulty encountered by readers when they are reading in the region [52].

Prior to all analyses, sentences with track losses were excluded (less than 2.5% of trials). In addition, fixations less than 80 ms in duration and within one character of the previous or subsequent fixation were incorporated into the neighbouring fixation. Remaining fixations of less than 80 ms were deleted, as were fixations of longer than 800 ms [53]. Finally, after means and standard deviations for the participants by condition, scoring region, and dependent measure had been computed, data greater than 3 *SD* from the condition mean were eliminated. Taken together, these procedures led to exclusion of 4.6% of the data. Four participants' data were removed because their reading comprehension accuracy rate was below 70%. As a result, only 56 participants' data remained valid.

For each measure in each region, we performed two ANOVAs, treating participants (*F*_1_) and items (*F*_2_) as random effects variables. In the participant analysis, the presence or absence of *huo zhe* at the beginning of the sentence and the presence or absence of temporary ambiguity were both treated as within-participants factors. In the items analysis, the presence or absence of *huo zhe* at the beginning of the sentence was a between-items factor, and the presence or absence of temporary ambiguity was a within-items variable. Table 1 presents the participants' means for each measure for each of the analysis regions, together with the standard deviations of these means.

**Table 1:** Participant mean reading times (in milliseconds) and percent regressions.

Measure	NP	Huo zhe NP	Verb
First fixation duration			
*huo zhe ambiguous S-coordination*	213 (35)	246 (47)	235 (79)
*huo zhe S-coordination*	221 (54)	242 (52)	236 (69)
*ambiguous S-coordination*	214 (67)	249 (82)	256 (82)
*S-coordination*	209 (45)	252 (61)	245 (84)
First pass time			
*huo zhe ambiguous S-coordination*	321 (63)	464 (110)	424 (116)
*huo zhe S-coordination*	315 (70)	455 (116)	418 (109)
*ambiguous S-coordination*	319 (80)	467 (125)	511 (193)
*S-coordination*	322 (72)	582 (104)	426 (123)
Go-past time			
*huo zhe ambiguous S-coordination*	418 (101)	633 (181)	673 (166)
*huo zhe S-coordination*	425 (123)	625 (129)	667 (149)
*ambiguous S-coordination*	434 (125)	635 (156)	795 (147)
*S-coordination*	417 (114)	779 (172)	675 (162)
Percent regressions			
*huo zhe ambiguous S-coordination*	3.7 (1.7)	6.06 (2.12)	9.07 (5.51)
*huo zhe S-coordination*	3.6 (1.9)	5.38 (2.08)	8.89 (4.79)
*ambiguous S-coordination*	4.3 (1.3)	6.12 (2.65)	28.13 (6.88)
*S-coordination*	3.1 (1.1)	20.03 (2.16)	9.53 (9.14)

*Note:* Standard deviations of the mean are presented in parentheses.

### NP region

The statistical analysis suggested that neither the effects of the sentence-initial *huo zhe* and temporary ambiguity nor the interaction of these two variables approached significance for any of the measures in this region (*F*s<.1, *p*s>.8). These results rule out the possibility that syntactic prediction has a general facilitative effect.

### Huo zhe NP region

No significant main effects of the sentence-initial *huo zhe* and temporary ambiguity on the first fixation duration measure were found. The interaction of these two variables did not reach significance (*F*s<.1, *p*s>.05).

The analysis of first pass time revealed significant main effects of the sentence-initial *huo zhe*, *F*_1_ (1, 55) = 7.09, *p*<.05, 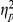 = .255, *F*_2_ (1, 22) = 6.78, *p*<.05, 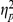 = .234, and of temporary ambiguity, *F*_1_ (1, 55) = 10.34, *p*<.01, 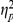 = .225, *F*_2_ (1, 22) = 9.34, *p*<.01, 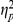 = .202. The interaction of these two variables was significant, *F*_1_ (1, 55) = 8.33, *p*<.01, 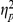 = .212, *F*_2_ (1, 22) = 5.23, *p*<.05, 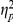 = .179. For the sentences preceded by *huo zhe*, the first pass time was more similar in ambiguous sentences and unambiguous sentences (464 ms vs. 455 ms). However, when the sentence-initial *huo zhe* was absent, the first pass time was approximately 115 ms shorter for *ambiguous S-coordination* than for *S-coordination*, *F*_1_ (1, 55) = 13.55, *p*<.001, 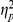 = .209, *F*_2_ (1, 22) = 11.87, *p*<.01, 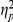 = .298.

Go-past time data yielded a main effect of the sentence-initial *huo zhe*, *F*_1_ (1, 55) = 26.06, *p*<.001, 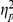 = .321, *F*_2_ (1, 22) = 22.15, *p*<.001, 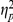 = .441. The main effect of temporary ambiguity was also significant. *F*_1_ (1, 55) = 5.73, *p*<.05, 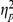 = .197, *F*_2_ (1, 22) = 12.06, *p*<.01, 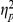 = .334. A significant interaction of these two variables was observed, *F*_1_ (1, 55) = 6.14, *p*<.05, 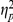 = .20, *F*_2_ (1, 22)  = 8.78, *p*<.01, 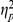 = .28. This interaction occurred due to a significant effect of temporary ambiguity in the absence of *huo zhe*, with longer go-past time for *S-coordination* than for *ambiguous S-coordination* (779 ms vs. 635 ms), *F*_1_ (1, 55) = 9.43, *p*<.01, 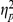 = .193, *F*_2_ (1, 22) = 18.13, *p*<.001, 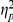 = .465. When the sentence-initial *huo zhe* was present, the go-past time did not differ significantly (625 ms vs. 633 ms), *F*s<.19, *p*s>.67.

The main effects of the sentence-initial *huo zhe* and temporary ambiguity were significant in the percentage regressions analysis (*F*s>5, *p*s<.05). The interaction of these two variables was significant, *F*_1_ (1, 55) = 5.07, *p*<.05, 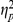 = .173, *F*_2_ (1, 22) = 3.86, *p*<.05, 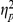 = .191. When *huo zhe* was absent at the beginning of the sentence, readers made more regressive eye movements for *S-coordination* than for *ambiguous S-coordination* (20.03% vs. 6.12%), *F*_1_ (1, 55) = 14.21, *p*<.01, 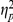 = .205, *F*_2_ (1, 22) = 13.51, *p*<.01, 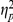 = .377. When the sentence-initial *huo zhe* was present, we did not observe this effect (*F*s<.6, *p*s>.44).

Overall, the pattern of data in the *huo zhe* NP region is quite clear. The presence of the sentence-initial *huo zhe* significantly reduced reading times (e.g., first pass time and go-past time) and regressions when readers processed the ambiguous and unambiguous regions. We observed an interaction between sentence type and the word *huo zhe*. In the absence of the sentence-initial *huo zhe*, readers spent more first pass time, go-past time, and regressions in the implausible NP region than in the plausible NP region. The first-pass time measure was an index of lexical processing and was sensitive to difficulty associated with syntactic disambiguation [47], [49]. The go-past time and regressions measures are often used to reflect the effect of syntactic reanalysis [54]. Thus, we believe that the absence of the sentence-initial *huo zhe* induced syntactic misanalysis and syntactic reanalysis of the implausible NP region. A speculative account is offered in the Discussion.

### Verb region

The first fixation duration results showed the significant effects of the sentence-initial *huo zhe*, *F*_1_ (1, 55) = 22.18, *p*<.001, 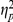 = .227, *F*_2_ (1, 22) = 20.66, *p*<.001, 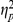 = .438, and of temporary ambiguity, *F*_1_ (1, 55) = 7.23, *p*<.05, 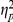 = .114, *F*_2_ (1, 22) = 14.25, *p*<.01, 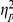 = .412. These two variables did not show a significant interaction (*F*s<1, *p*s>.1). Analysis of the first pass time showed main effects of the sentence-initial *huo zhe*, *F*_1_ (1, 55) = 7.27, *p*<.01, 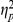 = .117, *F*_2_ (1, 22) = 4.78, *p*<.05, 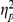 = .179, and of temporary ambiguity, *F*_1_ (1, 55) = 5.91, *p*<.05, 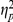 = .183, *F*_2_ (1, 22) = 9.23, *p*<.01, 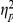 = .302. The interaction of these two variables was significant, *F*_1_ (1, 55) = 4.53, *p*<.05, 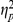 = .174, *F*_2_ (1, 22) = 7.45, *p*<.05, 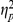 = .223. Tests for simple effects showed an effect of temporary ambiguity in the absence of the sentence-initial *huo zhe* (511 ms vs. 426 ms), *F*_1_ (1, 55)  = 8.13, *p*<.01, 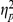 = .156, *F*_2_ (1, 22) = 17.01, *p*<.001, 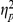 = .421. When the word *huo zhe* was present at the beginning of the sentence, the first pass time did not differ (424 ms vs. 418 ms), *F*s<.14, *p*s>.7. In addition, for the unambiguous sentences, sentence-initial *huo zhe* reduced the first-pass reading time (418 ms vs. 426 ms); however, this effect was not significant (*F*s<.39, *p*s>.53).

Analysis of the go-past time measure revealed significant main effects of the sentence-initial *huo zhe*, *F*_1_ (1, 55) = 15.37, *p*<.001, 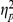 = .245, *F*_2_ (1, 22) = 7.98, *p*<.01, 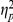 = .273, and of temporary ambiguity, *F*_1_ (1, 55) = 5.01, *p*<.05, 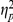 = .113, *F*_2_ (1, 22) = 6.13, *p*<.05, 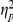 = .235. The interaction of these two variables was significant, *F*_1_ (1, 55) = 7.07, *p*<.05, 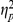 = .121, *F*_2_ (1, 22) = 5.34, *p*<.05, 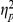 = .191. Tests of simple effects in the sentences without the sentence-initial *huo zhe* provided evidence for a temporary ambiguous effect (795 ms vs. 675 ms), *F*_1_ (1, 55) = 10.32, *p*<.01, 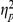 = .225, *F*_2_ (1, 22) = 11.42, *p*<.01, 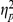 = .3334. The effect of temporary ambiguity was not significant when the word *huo zhe* was present at the beginning of the sentence (*F*s<.1, *p*s>.78). For the unambiguous sentences, the go-past time was shorter in the presence of the sentence-initial *huo zhe* than in its absence (667 ms vs. 675 ms), but this effect did not reach significance (*F*s<1.71, *p*s>.19).

The main effects of the sentence-initial *huo zhe* and of temporary ambiguity were significant in the percentage regressions analysis (*F*s>7, *p*s<.01). These two variables showed significant interaction, *F*_1_ (1, 55) = 5.23, *p*<.05, 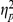 = .133, *F*_2_ (1, 22) = 7.02, *p*<.05, 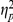 = .241. Simple effect analysis revealed a significant effect of temporary ambiguity (28.13% vs. 9.53%) only when the sentence-initial *huo zhe* was absent, *F*_1_ (1, 55) = 11.13, *p*<.01, 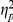 = .195, *F*_2_ (1, 22) = 18.23, *p*<.001, 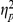 = .463. The effect of temporary ambiguity did not reach statistical significance when *huo zhe* was present (9.07% vs. 8.89%), *F*s<1.96, *p*s>.17. For the unambiguous sentence, the presence of the sentence-initial *huo zhe* reduced the regression percentages (8.89% vs. 9.53%); however, this effect was not significant (*F*s<1.24, *p*s>.27).

As previously noted, the first pass time was sensitive to syntactic misanalysis and the measures of go-past time and regressions were informative about syntactic reanalysis. With regard to the disambiguating region, we only observed the effects of reading time (e.g., first pass time and go-past time) and regressions when readers processed the *ambiguous S-coordination* structure in the absence of the sentence-initial *huo zhe*. Thus, we infer that when readers encountered the verb following the previous NP, they found that the previous coordinate structure was implausible. This incorrect syntactic analysis would induce a syntactic reanalysis.

## Discussion

In modern Chinese, *huo zhe* is one of the most important disjunctive conjunctions. The coordinate structure requires that the two sentence elements connected by *huo zhe* be the same grammatically (e.g., S or S, VP or VP, NP or NP). The results of this experiment show that the reading of critical regions was facilitated when *huo zhe* was presented in the sentence-initial position. In both ambiguous and unambiguous sentence-coordination structures, the presence of the sentence-initial *huo zhe* significantly reduced the first pass time, go-past time, and regressive eye movements in the regions containing the *huo zhe* NP and the verb. The presence of *huo zh*e eliminated the necessity of the NP-coordination analysis, which reduced the first pass time, go-past time, and regressive eye movements in the *huo zhe* region when the coordination was implausible and reduced those measures in the verb region when the coordination was plausible. In the *huo zhe* NP region, unambiguous coordination was associated with longer reading times (e.g., first pass time and go-past time) and more regressions than ambiguous coordination. In the verb region, syntactical disambiguation due to implausible NP-coordination analysis increased both reading times and regressions for the ambiguous coordination in comparison to the unambiguous coordination.

For native English speakers, extensive evidence indicates that noun phrase coordination analysis is preferred when reading sentences such as, *Either the boys will use the skis or the sled will make the deliveries* [25], [55], [56], [57]. The appearance of *will make* after *sled* allows for the NP-coordination analysis to be ignored. In this case, processing difficulty has appeared [55], [56], [58]. For native Chinese readers, the present experimental results strongly suggest an initial NP-coordination parsing preference. Absence of the sentence-initial *huo zhe* was connected to a cost related to abandoning the NP-coordination analysis. This cost affected the *huo zhe* NP region for the *S-coordination* condition and impacted the next syntactically disambiguating region for the *ambiguous S-coordination* condition.

Generally, we use terms from modularity and interactive processing to analyse the mechanisms of sentence comprehension. The modular view assumes that each factor involved in sentence processing is computed in its own module, which has limited means of communication with other modules. Interactive accounts assume that all available information is processed at the same time and can immediately influence the computation of the final analysis. Many previous studies have provided strong behavioural and electrophysiological evidence for the interactive account of sentence processing in Chinese [59], [60], [61], [62], [63]. For example, Peng and Liu (1993) used a self-paced reading paradigm to explore the relationship between syntax and semantics in Chinese sentence processing [61]. Participants were asked to make grammar decisions concerning each sentence, identifying it as plausible or implausible. Peng et al. found that the error rate difference between plausible and implausible sentences, both for ambiguous words and disambiguating words, was significant. Only the reaction time difference was significant for disambiguating words.

According to the interactive model, syntactic and semantic factors can influence sentence processing simultaneously [64]. Specifically, in the present experiment, the prediction of verbal structures was activated by the verb itself (e.g., *zi zhu*) before readers processed subsequent materials in the absence of the sentence-initial *huo zhe* [45]. Based on the analysis of verbal structures, readers likely inferred that because the verb was followed by a noun phrase, the NP-*huo zhe*-NP string was preferred. This preference enabled readers to adopt the NP-coordination analysis. However, when readers encountered the next verb in the syntactically disambiguating region, they had to abandon the initial NP-coordination analysis and conduct a syntactic reanalysis [65], [66], [67]. The present experimental data suggest that this reanalysis increased the first pass time, go-past time, and regressions. For the unambiguous sentences, readers found that the second NP could not serve as an object of the previous verb. This implausible semantic analysis immediately affected sentence processing. Therefore, the initial NP-coordination analysis was abandoned, and syntactic reanalysis occurred. The results show that readers spent more reading time and had more regressive eye movements in the implausible noun phrase regions than the plausible noun phrase regions. We note that Chen et al. (2010) obtained a series of effects with a converse pattern [37]. However, we argue that this inconsistency in results was due to some uncontrolled factors (e.g., poor experimental design, the number of strokes and the effect of the pronoun) in Chen et al.'s experiment. Judging from the present experiment, we believe that the noun phrase analysis is preferred when readers process coordinate structures in Chinese.

The second prediction regarding the present experiment was that the garden path effect would be eliminated when *huo zhe* preceded the entire sentence in Chinese. In this experiment, the word *huo zhe* connects two clauses. In half of the sentences, the subject in the second clause can be used as the object of the verb in first clause (for example, jingcha zhaodao wuzheng *huozhe* mujizhe, The police found physical evidence *or* the witnesses). In the other half of the sentences, the subject in the second clause cannot be used as the object of the verb in the first clause (for example, Lixiao yanchang jingju *huozhe* zuzhizhe, Li Xiao sings opera *or* concert organiser). When the verb in the second clause appears, the reader finds that the previous NP-coordination analysis is wrong and then activates the syntactic reanalysis programme. The pattern of the data showed that Chinese readers were likely to make regressive eye movements in ambiguous and disambiguating regions when the word *huo zhe* was absent at the beginning of the sentence. Importantly, the results demonstrated that when Chinese readers processed sentence-coordination structures, *huo zhe* at the beginning of a sentence significantly reduced reading times (e.g., first pass time and go-past time) and regressions in ambiguous and disambiguating regions. We believe that the most likely explanation for this effect in the *huo zhe* NP region and verb region is syntactic prediction. In Chinese, when the word *huo zhe* appears at the beginning of the sentence, the readers are able to predict that the sentence will contain a parallel structure. When Chinese readers find that the sentence-initial *huo zhe* is followed by a clause, they build a predictable syntactic structure (i.e., sentence-coordination structure) before encountering the corresponding lexical input and avoid the garden path effect induced by the initial NP-coordination analysis, as shown in Figure S1 (b) [1], [2], [3], [4], [21]. This top-down strategy eliminates the need to build sentence structure when readers reach this region. These data contrast with the results of an experiment conducted in English [25]. In the English experiment, Staub found that the presence of *either* did not help readers avoid the garden path effect and did not reduce the first pass reading time and the go-past time. We argue that this difference in findings may be due to the existence of commas in the English sentences. Many researchers have found that a comma at the end of a clause increases the number of regressive eye movements, saccade latencies, and fixations in the next region [23], [68]. As a result, the effect of the word *either* is likely to be enhanced or reduced.

Alternate accounts of the presented data should be considered. One may argue that the presence of the sentence-initial *huo zhe* may facilitate the processing of the entire sentence. In that case, we should have found an effect of the presence or absence of the sentence-initial *huo zhe* on reading times and regressive eye movements in the NP region before the second *huo zhe*. The absence of the effect, however, rules out this interpretation. A second explanation is that *huo zhe* presented at the beginning of the sentence has a facilitative effect because it predicts the use of another *huo zhe* before the second clause. Previous research has found that the preceding context reduces the reading times of highly predictable words [69], [70]. Moreover, the second *huo zhe* is always processed with eyes that were fixated on the previous region or during the first fixation of the next region [55]. In the present experiment, we did not find that the second *huo zhe* significantly reduced the duration of the first fixation. A third explanation is that the absence of *huo zhe* is associated with competition between syntactic alternatives, comparable to *either* in English [6], [71]. According to such an account, in Examples 5c and 5d, the S-coordination and NP-coordination are activated as soon as the *huo zhe* NP regions were in sight. This competition results in slower processing of the *huo zhe* NP region. We reject this explanation because, in the present study, the NP-coordination analysis was always implausible when readers processed *unambiguous S-coordination* sentences (e.g., *changzhang tigao daiyu huozhe gongchengshi jujue jixu qian xinde hetong*). This implausible NP-coordination increased reading times and regressions in the *huo zhe* NP region. When the NP-coordination analysis was plausible (e.g., *xiaozhang zizhu guer huozhe qitaren zuzhi qilai gongtong zizhu ta*), we found no hint of slower processing. These results indicate that a syntactic competition during the analysis of the coordination structures did not occur. Indeed, several studies have reported that ambiguity between syntactic alternatives does not slow processing [72], [73], [74]. We believe that syntactic reanalysis is the main reason for the increased reading times and regressions in the *huo zhe* NP region during the *S-coordination* in the absence of a sentence-initial *huo zhe*. In 5d, for example, readers could not build a predictable syntactic structure. They sometimes analysed the word *gong cheng shi* as the direct object of the word *ti gao*. The implausibility of the VP caused processing difficulty and increased reading times. Readers had to make more regressive eye movements. When the sentence was preceded by *huo zhe*, readers were less likely to conduct this incorrect analysis. In addition, reading times and regressive eye movements did not significantly increase for *ambiguous S-coordination*, given that the subject of the second clause could serve as the direct object of the previous verb (e.g., *zizhu guer huozhe qitaren* in Example 5c).

In summary, the critical finding of this study is the existence of an initial NP-coordination parsing preference in Chinese sentence-coordination structure. Whenever *huo zhe* was absent, we identified a cost associated with abandoning the NP-coordination analysis. This cost appeared in the second NP when the coordination was unambiguous and emerged in the next syntactically disambiguating region when the coordination was ambiguous. In addition, syntactic prediction is likely to have a facilitative effect. When presented in the sentence-initial position, *huo zhe* removes, or at least reduces the implausible NP-coordination analysis and renders an *S-coordination* relatively predictable before encountering the linguistic input.

## Supporting Information

Materials S1 **Experimental items.**
(DOC)

Figure S1 **Postulated stages in the parser's analysis of coordinate sentences.**
(TIF)
