# Supplementary material for: Processing Coordinate Structures in Chinese: Evidence from Eye Movements
Source: PLoS One. 2012 Apr 25;7(4):e35517. doi: 10.1371/journal.pone.0035517 (PMC3338849; doi:10.1371/journal.pone.0035517)
Supplement: Materials S1 — Experimental items. (DOC) [file pone.0035517.s001.doc]

**Experimental items**

**Unambiguous sentences**

1. (或者) 孙玉借到菜刀或者老板娘跑到商店买一把新的。

(Either) Sun Yu borrows a knife or the landlady runs to the store to buy a new one.

2. (或者) 厂长提高待遇或者工程师拒绝继续签新的合同。

(Either) the factory director improves the treatment or the engineers refuse to sign the new contract.

3. (或者) 李晓演唱京剧或者组织者安排别的人出来表演。

(Either) Li Xiao sings Beijing Opera or the organisers arrange for others to perform.

4. (或者) 张敏撰写报告或者副市长抽空亲自写这份报告。

(Either) Zhang Min writes this report or the mayor finds the time to write this report.

5. (或者) 汪东打扫讲台或者班主任拿出湿抹布打扫讲台。

(Either) Wang Dong cleans the rostrum or the head-teacher uses a damp rag to clean the rostrum.

6. (或者) 刘伟重盖房子或者儿女们出钱为他买套新房子。

(Either) Liu Wei rebuilds this house or his children buy him a new one.

7. (或者) 叶林买辆汽车或者儿媳妇租借一辆汽车给她用。

(Either) Ye Lin buy a car or his daughter-in-law rents a car for her.

8. (或者) 钱月扮演学生或者总导演重新给她找一个角色。

(Either) Qian Yue acts as a student or the chief director assigns her a new role.

9. (或者) 赵明出任秘书或者课题组挑选别的人来做秘书。

(Either) Zhao Ming works as the secretary or the research team chooses another person as the secretary.

10. (或者) 王刚放弃学业或者抚养人借到钱让他继续读书。

(Either) Wang Gang drops out of school or his custodian lends him money to support his studies.

11. (或者) 学校提供午饭或者副乡长邀请老师到酒店吃饭。

(Either) the school provides teachers with lunch or the village head invites teachers to a restaurant for lunch.

12. (或者) 杨月承担损失或者开发商赔偿违约造成的损失。

(Either) Yang Yue bears the loss or the developer compensates for the loss caused by the violation of contracts.

**Ambiguous sentences**

1. (或者) 校长资助孤儿或者其他人组织起来共同资助他。

(Either) the headmaster supports the orphan or other people organise together to support him.

2. (或者) 工厂补贴工人或者管理者废除周末加班的制度。

(Either) the factory compensates workers or the manager abolishes the working-on-weekends system.

3. (或者) 吴伟代表公司或者总经理选择一个更有资历的。

(Either) Wu Wei represents the company or the general manager chooses another more qualified person.

4. (或者) 张杰照顾妻子或者孩子们花钱雇个保姆照顾她。

(Either) Zhang Jie takes care of his wife or the children hire a nursemaid to look after her.

5. (或者) 林东看望厂长或者董事长吩咐别人来做这件事。

(Either) Lin Dong pays the factory director a visit or the chairman orders others to do this.

6. (或者) 警察找到物证或者目击者愿意为受害者当人证。

(Either) the police found physical evidence or the witnesses are willing to testify for the victims.

7. (或者) 陈希起诉商家或者乡政府协助他讨回所有损失。

(Either) Chen Xi prosecutes the businessman or the town government helps him to get his loss back.

8. (或者) 老师辅导差生或者好学生帮助这些人补习功课。

(Either) the teachers tutor lagged students or the excellent students help them with their studies.

9. (或者) 学校惩罚班长或者辅导员代替他承担所有责任。

(Either) the school punishes the monitor or the instructor takes responsibility for him.

10. (或者) 主人解雇佣人或者老管家出面求情争取留下她。

(Either) the master fires the servant or the old housekeeper intercedes for her.

11. (或者) 李娜担任队长或者团支书推荐别的同学代替她。

(Either) Li Na takes charge of the team or the league branch secretary recommends other students to replace her.

12. (或者) 镇长撤掉村长或者村支书找到替罪羊帮他顶罪

(Either) the town mayor fires the village mayor or the village party secretary finds a scapegoat for him.
