# Supplementary figures and images for: Processing Coordinate Structures in Chinese: Evidence from Eye Movements
Source: PLoS One. 2012 Apr 25;7(4):e35517. doi: 10.1371/journal.pone.0035517 (PMC3338849; doi:10.1371/journal.pone.0035517)

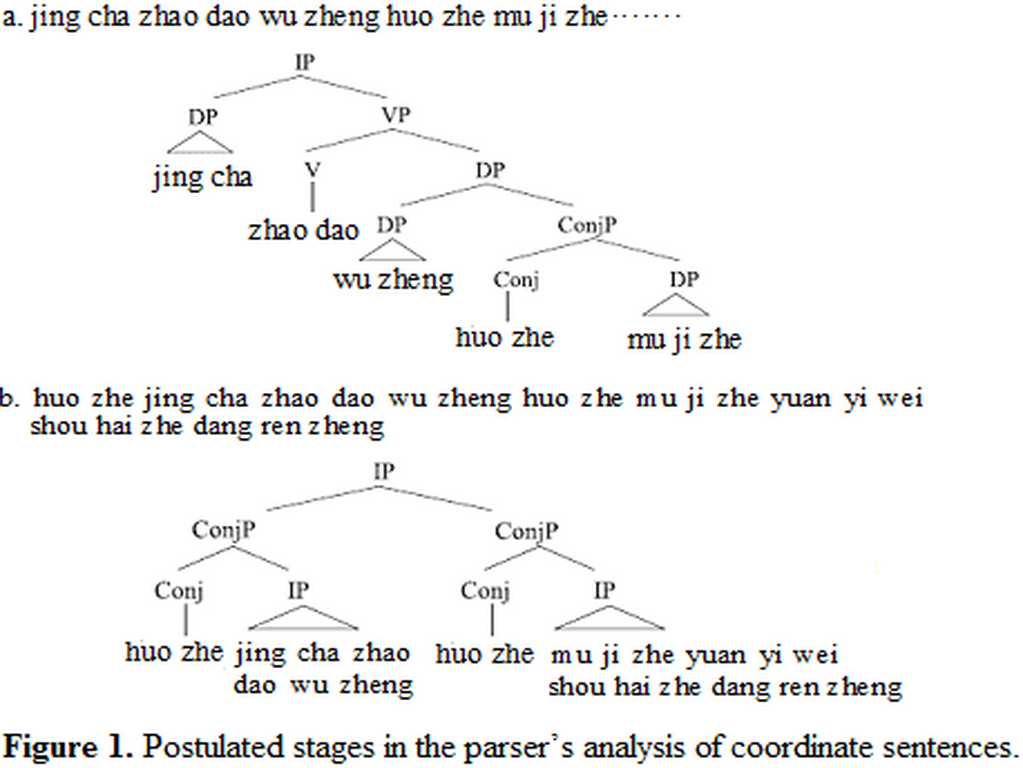

Supplement: Figure S1 — Postulated stages in the parser's analysis of coordinate sentences. (TIF) [file pone.0035517.s002.tif]
